# Representation and Characterization of Nonstationary Processes by Dilation Operators and Induced Shape Space Manifolds

**DOI:** 10.3390/e20090717

**Published:** 2018-09-19

**Authors:** Maël Dugast, Guillaume Bouleux, Eric Marcon

**Affiliations:** Univ Lyon, UJM-Saint Etienne, INSA Lyon, DISP, F-69621 Villeurbanne, France

**Keywords:** nonstationary processes, spectral measure, differential geometry, shape manifold, square root velocity function, Lie group

## Abstract

We proposed in this work the introduction of a new vision of stochastic processes through geometry induced by dilation. The dilation matrices of a given process are obtained by a composition of rotation matrices built in with respect to partial correlation coefficients. Particularly interesting is the fact that the obtention of dilation matrices is regardless of the stationarity of the underlying process. When the process is stationary, only one dilation matrix is obtained and it corresponds therefore to Naimark dilation. When the process is nonstationary, a set of dilation matrices is obtained. They correspond to Kolmogorov decomposition. In this work, the nonstationary class of periodically correlated processes was of interest. The underlying periodicity of correlation coefficients is then transmitted to the set of dilation matrices. Because this set lives on the Lie group of rotation matrices, we can see them as points of a closed curve on the Lie group. Geometrical aspects can then be investigated through the shape of the obtained curves, and to give a complete insight into the space of curves, a metric and the derived geodesic equations are provided. The general results are adapted to the more specific case where the base manifold is the Lie group of rotation matrices, and because the metric in the space of curve naturally extends to the space of shapes; this enables a comparison between curves’ shapes and allows then the classification of random processes’ measures.

## 1. Introduction

**Figure 1:**
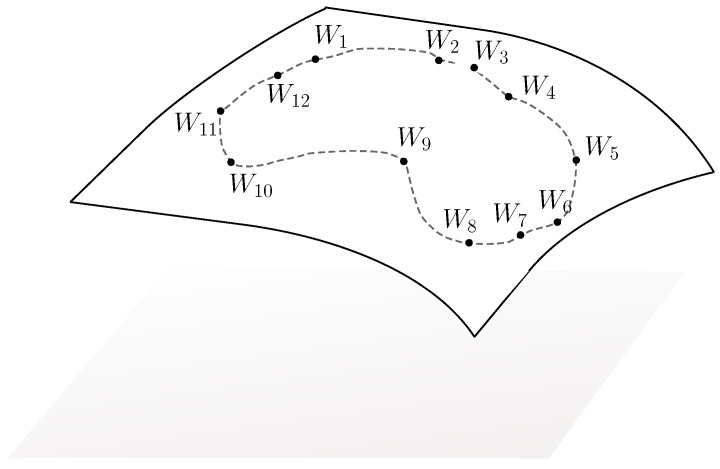
Illustration of a sampled closed trajectory drawn in SO(n) or SU(n) that materializes the time varying of the Periodically Correlated (PC) measure for a stochastic process. Each $W_{i}$ is a dilation matrix built through the parcors. Recall that a PC process is a process such that $R_{s,t}=R_{s+T,t+T}$ for a certain T, where $R_{\cdot ,\cdot }$ stands for the correlation function of the process.

The analysis and/or the representation of nonstationary processes has been tackled for four or five decades now by time-scale/time-frequency analysis [1,2], by Fourier-like representation when the processes belong to the periodically correlated (PC) subclass [3,4], or by partial correlation coefficients (parcors) series [5,6], to cite a few. One of the advantages of dealing with parcors resides in their strong relation to the measure of the process by the one-to-one relation with correlation coefficients [7,8]. They consequently appear explicitly in the Orthogonal Polynomial on the Real Line/Unit Circle decomposition of the measure [9,10], on the Matrices Orthogonal Polynomials on the Unit Circle [11] and its applications [12], are the elements for the construction of dilation matrices that appear in the Cantero Moral, and Velazquez (CMV)/Geronimus, Gragg, and Teplyaev (GGT) matrices [13], for the Schur flows problem with upper Hessenberg matrices [14] that are also seen in the literature as evolution operators [10] or shift operator [15], and finally appear in the state-space representation [7,16]. The dilation theory takes its roots from the operator theory [17], which bridges the process’s measure and unitary operators. In its simplest version, the dilation theory corresponds to Naimark dilation [17,18], and states that given a sequence of correlation coefficients, there exists a unitary matrix *W* such that $R_{n}≜\left(1\;0\;0\cdots \right)W^{n}{\left(1\;0\;0\cdots \right)}^{T}$ where $\cdot^{T}$ denotes the transposition. When the process is not stationary, its associated correlation matrix is no more Toeplitz structured, a set of matrices is required [16] and the previous expression becomes $R_{i,j}≜\left(1\;0\;0\cdots \right)W_{i+1}W_{i+2}\cdots W_{j}{\left(1\;0\;0\cdots \right)}^{T}$. The matrices $W_{i}$ are theoretically understood as infinite rotation matrices, which become finite when the correlation coefficients sequence is itself finite. In that particular case, the matrices $W_{i}$ belong to SO(n) or SU(n), the special orthogonal or unitary group, respectively, and the process’s measure is totally described by the set of $W_{i}$. As a consequence, the measure of the process is beautifully characterized for the nonstationary case, by a sampled trajectory induced by the dilation matrices on the appropriate Lie group. When the process is periodically correlated, the sequence of parcors is periodic, as well as the sequence of dilation matrices, which yields a closed path as illustrated in Figure 1. This raises the question of comparison of processes by means of their dilation matrices. Many efforts have been made in the last decade to exploit the hyperbolic geometric structure not of the correlation matrices directly but of the related parcors when obtained in stationary conditions [8,19,20,21,22], as well as the information geometry structure that is closely related to it [23,24,25]. As the Kullback–Leibler divergence let do, the comparison of stationary processes is then made by comparing curves, whose sampled points are parcors sequences, defined on several copies of the Poincaré disk through geodesics deformation. Treatment of the nonstationary case has not been tackled to our knowledge with the previously mentioned approaches. In this paper, we hope to initiate interest in filling this gap by extending the representation and the characterization of the processes’s measure in a nonstationary context, inspired on the one hand by information geometric application and interpretation of parcors [19,20] or correlation matrices [26,27,28], and on the other hand by theories and applications dealing with curves on manifolds [29,30], closely related to some aspects of Euler’s equations [31]. Therefore, we combine Constantinescu’s approach to dilation and shape analysis to the propose of seeing stochastic processes as elements of a Lie group. Characterizing the time-varying measure of the process is now tackled by studying curves (or sampled curves) on special groups.

To support the reader, some insights on dilation theory are given in Section 2. Practical implementations of dilation matrices according to the operator theory approach [16,18] or the lattice filter structure approach [32,33] are also discussed and the strong connection between parcors and the dilation matrices is emphasized. Section 3 focuses on the geometry of the curves induced by the dilation on particular manifolds. The general framework is first introduced by recalling concepts of distances and shape of curves when the ambient space is not flat. Next, the square root velocity (SRV) functions are developed and adapted to the Lie group, and a procedure to compare nonstationary processes through their time evolution trajectory is presented. Finally, a conclusion is drawn in Section 4 and the reader will find some technical tools in Appendix A and Appendix B.

## 2. The Structure of Semi-Positive-Definite Matrices and the Dilation Theory

### 2.1. Outline of the Dilation Theory

Let us give some insights into the dilation theory. In its fundamental definition, the dilation theory consists of a Hilbert space H and an operator-valued function *f*, i.e., an L(H)-valued function, to find a larger Hilbert space *H* and another application F such that *f* is the orthogonal projection of F:

(1) $$f\left(t\right)=P_{H}F\left(t\right),\;t\in Z$$

where $P_{H}$ denotes the orthogonal projection onto the Hilbert space H. The ideas of the dilation theory are:

- there exists a larger space from which the original function (or matrix) is deduced;
- we can choose the “dilated” function to be simpler. For instance, when dealing with matrices, each of its coefficients can be expressed as the projection of a larger unitary matrix. In this case, we obtain a unitary dilation. This approach has been for example developed in [34,35,36] for the stationary dilation of periodically-correlated processes.

#### 2.1.1. Dilation and Rotation of Contractions

For an operator *T* on a Hilbert space H, we denote by $T^{*}$ the adjoint operator, i.e., the operator on H such that $\langle Tx,y\rangle =\langle x,T^{*}y\rangle$ for all x,y∈H. An operator T∈L(H) is said to be a contraction if ∣∣T∣∣≤1 where ∣∣·∣∣ is the operator norm. We deduce the expression for the defect operator $D_{T}={\left(I-T^{*}T\right)}^{1/2}$ and its adjoint $D_{T^{*}}={\left(I-TT^{*}\right)}^{1/2}$.

One of the easiest results is that, given a contraction Γ, the following unitary operator

(2) $$J\left(\Gamma \right)=\left(\begin{array}{cc} \Gamma & D_{\Gamma^{*}}\\ D_{\Gamma } & -\Gamma^{*} \end{array}\right)$$

satisfies, for all n∈N

(3) $$\Gamma^{n}=\left(\begin{array}{cc} 1 & 0 \end{array}\right)J{\left(\Gamma \right)}^{n}\left(\begin{array}{c} 1\\ 0 \end{array}\right).$$

In other words, the elementary rotation of a contraction also corresponds to the unitary dilation operator of the contraction. This operator is called the Julia operator, and corresponds to the Halmos extension [15] of a contraction.

#### 2.1.2. Dilation and Isometries

Following the idea and the formulation of Naimark, the dilation theory can be restated in terms of dilation of the sequence of operators or sequence of numbers when the dimension of the underlying Hilbert space is 1. A sequence of operators ${\left\{R_{n}\right\}}_{n=1}^{\infty }$ acting on H is said to be positive if

(4) $$\sum_{i,j=0}^{+\infty }\langle R_{i-j}h_{i},h_{j}\rangle \geq 0\;\mathrm{for}\;\mathrm{all}\;h_{i}\in H_{i}.$$

Assuming now that $R_{n}^{*}=R_{-n}$ and $R_{0}=I$, leads to the following Toeplitz matrix:

(5) $$R^{\left(m\right)}=\left(\begin{array}{cccc} I & R_{1} & \cdots & R_{m-1}\\ R_{1}^{*} & I & \cdots & R_{m-2}\\ \cdot & \cdot & \cdots & \cdot \\ \cdot & \cdot & \cdots & \cdot \\ R_{m-1}^{*} & R_{m-2}^{*} & \cdots & I \end{array}\right)$$

which is positive-definite. Remark that this matrix can be seen as the correlation matrix of a stationary process, as it is positive and Toeplitz [37]. Owing to this property, we obtain the existence of an operator *U* such that [18,38] and Theorem 1.1 in [39]:

(6) $$R_{n}=P_{H}U^{n}{|}_{H},\;\mathrm{for}\;\mathrm{all}\;n\geq 0\;\;\mathrm{and}\;\;U\;\mathrm{an}\;\mathrm{isometry}\;\mathrm{on}\;K$$

as a result of the Naimark dilation theorem [17]. Furthermore, if $K=\underset{n\geq 0}{⋁}U^{n}H$, where ⋁ denotes the linear span, then *U* is unique up to an isomorphism.

#### 2.1.3. Dilation and Measure

From Bochner’s theorem [37], we know a matrix of type (5) can be seen as the Fourier coefficient of a given positive Borelian measure. This is also known as the moment or trigonometric problem [16]. Therefore, we can restate the dilation problem in terms of measure. If we denote by $E_{\lambda }$ an operator-valued distribution function on [0,2π], then the function

(7) $$R_{n}=\int_{0}^{2\pi }e^{in\lambda }dE_{\lambda }.$$

is positive-definite. This shows the strong correspondence between the spectral measure and the dilation theory. There hence exists a unitary operator on a Hilbert space K such that $R_{n}=P_{H}U\left(n\right)$ where $P_{H}$ stands for the orthogonal projection. With the spectral representation of unitary operators, $U=\int_{0}^{2\pi }e^{i\lambda }dE_{\lambda }$ and we have

(8) $$\int_{0}^{2\pi }e^{in\lambda }d\langle E_{\lambda }u,v\rangle =\int_{0}^{2\pi }e^{in\lambda }d\langle F_{\lambda }u,v\rangle$$

or, in an equivalent form:

(9) $$E_{\lambda }=P_{H}F_{\lambda }.$$

Note that the operator-valued measure $F_{\lambda }$ is in fact an orthogonal projection-valued measure because all its increments are orthogonal. With dilation matrices having been introduced, we give in the next section a methodology to understand how they are obtained.

### 2.2. Construction of Dilation Matrices

As mentioned previously, given an SPD matrix $R={\left(R_{i,j}\right)}_{i,j\in N}$, it is possible to build a sequence of matrices ${\left\{W_{i}\right\}}_{i\in N}$ such that $R_{i,j}=\left(\begin{array}{ccccc} 1 & 0 & 0 & \cdots & 0 \end{array}\right)W_{i}W_{i+1}\cdots W_{j-1}{\left(\begin{array}{ccccc} 1 & 0 & 0 & \cdots & 0 \end{array}\right)}^{T}.$ Let the general framework where $R_{i,j}$ is a complex operator satisfying $R_{i,j}\in L\left(H_{j},H_{i}\right)$ with ${\left\{H_{n}\right\}}_{n}$ a sequence of Hilbert spaces and L the set of linear applications. For example, consider the stochastic process ${\left\{X_{n}\right\}}_{n}$, where $X_{n}\in L^{2}\left(P\right)$ is a squared integrable random variable with respect to the probability space $\left(\Omega ,F,P\right)$. Then the stochastic process can be viewed as an operator: ${\tilde{X}}_{n}:C\to L^{2}\left(P\right),{\tilde{X}}_{n}\lambda =\lambda X_{n}$, and the correlation kernel becomes $R_{i,j}={\tilde{X}}_{i}^{*}{\tilde{X}}_{j}$.

To start the construction, let first the following theorem

**Theorem** **1** (Structure of a positive-definite block matrix)**.** *Let X and Z be positive operators in $L(H_{X})$ and $L(H_{Z})$ respectively. Then the following are equivalent :*

- The operator $A=\left(\begin{array}{cc} X & Y\\ Y^{*} & Z \end{array}\right)$ is positive
- *There exists a unique contraction *Γ* in L(R(Z),R(X)) such that*
  (10) $$Y=X^{1/2}\Gamma Z^{1/2}$$

**Proof.**  Appendix A ☐

Let us now apply this relation repeatedly on an SPD matrix. To fix ideas, let the *R* be the 3×3 (block-)matrix:

(11) $$R=\left(\begin{array}{ccc} R_{1,1} & R_{1,2} & R_{1,3}\\ R_{1,2}^{*} & R_{2,2} & R_{2,3}\\ R_{1,3}^{*} & R_{2,3}^{*} & R_{3,3} \end{array}\right)$$

and apply Theorem 1 to $\left(\begin{array}{cc} R_{1,1} & R_{1,2}\\ R_{1,2}^{*} & R_{2,2} \end{array}\right)$, $\left(\begin{array}{cc} R_{2,2} & R_{2,3}\\ R_{2,3}^{*} & R_{3,3} \end{array}\right)$ and finally to $\left(\begin{array}{cc} R_{1,2} & R_{1,3} \end{array}\right)$. Note that when a square root of a (block-)matrix has to be chosen, it is done according to the Schur decomposition given in Appendix A. For example, we have $R_{1,2}=R_{1,1}^{1/2}\Gamma_{1,2}R_{1,1}^{1/2}$. At each step, a contraction $\Gamma_{i,j}$ is generated with respect to the indices of the upper and lower (block-)matrices of the main diagonal, e.g., $\Gamma_{1,2}$ for the first $\left(\begin{array}{cc} R_{1,1} & R_{1,2}\\ R_{1,2}^{*} & R_{2,2} \end{array}\right)$ (block-)matrix. We thus obtain a one-to-one correspondence between the SPD matrix *R* and the set of contractions ${\left\{\Gamma_{i,j}\right\}}_{i=1,2\;j=3}$. Regarding the huge work of Constantinescu [16], we will call these contractions the Schur-Constantinescu parameters. We consider now unit variance and arbitrary size n×n for the SPD matrix, which allows us to write the correspondence as:

(12) $$\left(\begin{array}{ccc} R_{1,1} & R_{1,2} & R_{1,n}\\ R_{1,2}^{*} & R_{2,2} & \ddots \\ \ddots & \ddots & R_{n-1,n}\\ R_{1,n}^{*} & R_{n-1,1}^{*} & R_{n,n} \end{array}\right)⟷\left(\begin{array}{cccccc} 0 & \Gamma_{1,2} & \Gamma_{1,3} & \cdots & \Gamma_{1,n}\\ 0 & 0 & \Gamma_{2,3} & \Gamma_{2,4} & \cdots & \Gamma_{2,n}\\ \vdots & \ddots & \ddots & \ddots \\ \Gamma_{n-2,n}\\ 0 & \Gamma_{n-1,n}\\ 0 & 0 & \cdots & 0 \end{array}\right).$$

Once (12) is established, each dilation matrix $W_{i}$ is built-up as a product of Givens rotations of a sequence of Schur-Constantinescu parameters in the following way:

(13) $$W_{i}=G\left(\Gamma_{i,i+1}\right)G\left(\Gamma_{i,i+2}\right)\cdots G\left(\Gamma_{i,j}\right),$$

where $G_{\Gamma_{i,i+l}}$ denotes the Givens rotation of $\Gamma_{i,i+l}$ as follows:

(14) $$G\left(\Gamma_{i,i+l}\right)=I⊕\left(\begin{array}{cc} \Gamma_{i,i+l} & D_{\Gamma_{i,i+l}^{*}}\\ D_{\Gamma_{i,i+l}} & -\Gamma_{i,i+l}^{*} \end{array}\right)⊕I=\left(\begin{array}{cccccccc} 1 & 0 & 0 & 0 & 0 & \cdots & 0\\ 0 & 1 & 0 & 0 & 0 & \cdots & 0\\ \ddots & 0\\ 0 & \cdots & 0 & \Gamma_{i,i+l} & D_{\Gamma_{i,i+l}^{*}} & 0 & \cdots & 0\\ 0 & \cdots & 0 & D_{\Gamma_{i,i+l}} & -\Gamma_{i,i+l}^{*} & 0 & \cdots & 0\\ 0 & \cdots & 0 & 0 & 1 & 0 & \cdots & 0\\ \vdots & \ddots & \vdots \\ 0 & \cdots & 1 \end{array}\right)$$

where the “non-identity” part, consisting of a Julia operator $\left(D_{\Gamma_{i,i+l}}={\left(I-\Gamma_{i,i+l}^{*}\Gamma_{i,i+l}\right)}^{1/2}\right)$ is located at the entry (i,i). When the SPD matrix is Toeplitz, which corresponds to a stationary underlying process, then all dilation matrices $W_{i}$ are identical and take the form

(15) $$W_{i}=U=\left(\begin{array}{ccccc} \Gamma_{1} & D_{\Gamma_{1}^{*}}\Gamma_{2} & D_{\Gamma_{1}^{*}}D_{\Gamma_{2}^{*}}\Gamma_{3} & D_{\Gamma_{1}^{*}}D_{\Gamma_{2}^{*}}D_{\Gamma_{3}^{*}}\Gamma_{4} & \cdots \\ D_{\Gamma_{1}} & -\Gamma_{1}^{*}\Gamma_{2} & -\Gamma_{1}^{*}D_{\Gamma_{2}^{*}}\Gamma_{3} & -\Gamma_{1}^{*}D_{\Gamma_{2}^{*}}D_{\Gamma_{3}^{*}}\Gamma_{3} & \cdots \\ 0 & D_{\Gamma_{2}} & -\Gamma_{2}^{*}\Gamma_{3} & -\Gamma_{2}^{*}D_{\Gamma_{3}^{*}}\Gamma_{4} & \cdots \\ 0 & 0 & D_{\Gamma_{3}} & -\Gamma_{3}^{*}\Gamma_{4} & \cdots \\ 0 & 0 & 0 & D_{\Gamma_{4}} & \cdots \\ \cdot & \cdot & \cdot & \cdot & \cdots \\ \cdot & \cdot & \cdot & \cdot & \cdots \end{array}\right)$$

which is nothing less than the Naimark dilation introduced in the first part, i.e., $R_{i,j}=R_{j-1}=\left[1\;0\;0\;\cdots \right]U^{j-i}{\left[1\;0\;0\;\cdots \right]}^{T}$. For the sake of completeness, we give the correspondence between the coefficients of the SPD matrix (the left-hand side of (12)) and the Schur-Constantinescu parameters:

**Theorem** **2.** The matrix $R^{\left(n\right)}={\left[R_{k,j}\right]}_{k,j=1}^{n}$, satisfying $R_{j,k}^{*}=R_{k,j}$ is positive if and only if

- *$R_{kk}⩾0$ for all k*
- *there exists a family $\left\{\Gamma_{k,j}|k,j=1,\cdots n,k⩽j\right\}$ of contraction such that*
  (16) $$R_{k,j}=B_{k,k}^{*}\left(L_{k,j-1}U_{k+1,j-1}C_{k+1,j}\;+\;D_{\Gamma_{k,k+l}^{*}}\cdots D_{\Gamma_{k,j-l}^{*}}\Gamma_{k,j}D_{\Gamma_{k+1,j}}\cdots D_{\Gamma_{j-1,j}}\right)B_{j,j}$$
  *where $B_{k,k}$ is the Cholesky’s square-root of $R_{k,k}$.*

*and*

(17) $$L_{k,j}=\left[\Gamma_{k,k+1}\;D_{\Gamma_{k,k+l}^{*}}\Gamma_{k,k+2}\;\cdots \;D_{\Gamma_{k,k+l}^{*}}\cdots D_{\Gamma_{k,j-1}^{*}}\Gamma_{k,j}\right]$$

*a row contraction associated to the set of parameters $\left\{\Gamma_{k,m}|k<m\leq j\right\}$,*

(18) $$C_{k,j}={\left[\Gamma_{j-1,j}\;\Gamma_{j-2,j}D_{\Gamma_{j-1,j}}\;\cdots \;\Gamma_{k,j}D_{\Gamma_{k+1,j}}\cdots D_{\Gamma_{j-1,j}}\right]}^{T}$$

*a column contraction associated to the set of parameters $\left\{\Gamma_{m,j}|m=j-1,\cdots k\right\}$, and finally*

(19) $$U_{k,j}=G\left(\Gamma_{k,k+1}\right)G\left(\Gamma_{k,k+2}\right)\cdots G\left(\Gamma_{k,k+j}\right)\left(U_{k+1,j}⊕I\right)$$

**Proof.**  This theorem is proved in ([16], Theorem 5.3). ☐

A different approach leading to the same results can be found in [40], using directly the Kolmogorov decomposition. In [32] the Naimark dilation is constructed using the lattice filter and finally applications of this decomposition in quantum mechanics are to be found in [41,42] for example.

We now give some remarks to conclude this part:

- If $R^{\left(n\right)}$ is a semi-positive definite complex-valued Toeplitz kernel, then all the $\left\{\Gamma_{n}\right\}$ are complex-valued and respect $|\Gamma_{i}|<1$. The structure and the construction procedure for obtaining such a complex-valued parameter is identical whether the kernel is real or complex.
- The framework proposed by Constantinescu and recalled previously is quite general and can be extended to Matrix Orthogonal Polynomial on the Unit Circle (MOPUC) development. By referring to ([43], Section 3.1), matrix polynomials stem from a Szëgo recursion akin to the scalar case and thus provide a sequence of Verblunsky coefficients, or parcors that are matrices. Again in ([43], Section 3.11), a correspondence between MOPUC and CMV matrices [13] is provided, which are equivalent to dilation matrices. The construction procedure remains the same for the dilation matrices, but the parcors become in that case matrices (they are matrix-valued Verblunsky coefficients), which can be obtained by a matrix version of the Schur/Geronimus algorithm [44].

Dilation matrices being now fully introduced, we focus the attention of the reader on the hidden information contained in their timely geometrical dissemination.

## 3. Analysis of Curves on a Manifold Induced by the Dilation

Parcors, composing dilation matrices, have already been given a geometrical point of view, as, for example, in [8] where the sequence of parcors associated with a stationary process is seen as a point onto the Poincaré polydisk $P^{n}$, that is, the product of the Poincaré disk. To give geometrical settings, a distance to characterize individual parcors is then proposed and discussed. In [45], a stochastic process is studied under the local stationarity assumption. To each stationary slice of the process corresponds a sequence of parcors, represented as a point in the Poincaré polydisk $P^{n}$ as well. A trajectory is then generated on that space which materializes a curve on the manifold $P^{n}$. The underlying computations are quite intricate because of the product manifolds, and the question of nonstationarity arises. Based on the works of Le Brigant [45,46], Celledoni et al. [47] and Zhang et al. [48], we propose then to give particular attention to this question. We first make use of the dilation theory introduced in Section 2. When the process under study is nonstationary, a set of matrices $W_{i}$ is obtained. The basic idea for having geometric information on the nonstationary process is therefore to characterize the trajectory formed by the set of dilation matrices. These matrices are theoretically operators of infinite dimension, but as we dispose of only a finite set of parcors, the theoretical matrices of (15) are truncated. Matrices respecting (15) are general rotation matrices that become perfect rotation operators belonging to SO(n) for real processes and SU(n) when dealing with complex processes, when their dimensions are reduced to n×n. Our aim is finally to analyse those curves living on the Lie group of rotation matrices and emphasize the geometry or, more precisely, the intrinsic geometry formulation of these objects. For example, we aim at comparing different curves coming from different processes or at resuming many realizations of a stochastic process (multiple measurements) through the computation of the mean of the associated several curves. The question as to computation complexity still exists, but many results have been proposed recently to overcome this difficulty and to propose closed-form formulations [30,49]. In particular, it is predicated to extract the shape of the trajectory for it contains the essential information, in a topologic sense.

To allow the curves comparison, we have based our development on the works of Le Brigant [45] and Celledoni et al. [47]. First, we define the manifold M given by the set of all curves in the base manifold. This leads to another space, the shape space, for which the manifold M will be a principal bundle. We dispose then of a metric in M from which a metric on the shape space is deduced. These steps are now explained in the following.

### 3.1. Preliminaries on Lie Groups

As we are going to deal with curves on a Lie group, we start with some preliminaries.

A metric 〈·,·〉 on a Lie group is said to be left invariant if:

(20) $${\langle u,v\rangle }_{b}={\langle {\left(dL_{a}\right)}_{b}u,{\left(dL_{a}\right)}_{b}v\rangle }_{ab}$$

where ${\left(dL_{a}\right)}_{b}$ is the derivative in the manifold field sense (so the tangent map) of the left translation $L_{a}$ at *b*. A left-invariant metric gives the same number whenever the vectors are translated on the left. It is straightforward to adapt this definition to a right-invariant metric. A metric that is both left and right invariant is called a bi-invariant metric. A Lie group endowed with a bi-invariant metric has plenty of import properties that can be exploited for our study of curves on shape spaces. We list some of them in the following [50].

- The geodesics through *e* (the identity element) are the integral curves t↦exp(tu),u∈g, that is, the one-parameter groups. In addition, because left and right are isometries and isometries maps geodesics to geodesics, the geodesics through any point a∈G are the left (right) translates of the geodesics through *e*
  (21) $$\gamma \left(t\right)=L_{a}\left(exp(tu)\right),\;u\in g.$$
  Of course, we have
  (22) $$\gamma^{\prime }\left(0\right)=\left(dL_{a}\right)e\left(u\right).$$
- The Levi-Civita connection is given by : $\nabla_{X}Y=\frac{1}{2}\left[X,Y\right],\;\forall X,Y\in g$

where [·,·] denotes the Lie bracket. We can now link these formulas to our base manifold SO(n). The Killing form, *B*, of a Lie algebra is the symmetric bilinear form B:g×g⟶C given by B(u,v)=tr(ad(u)∘ad(v)), where tr denotes the trace operator and ad denotes the adjoint representation of the group, namely, the map ad:G⟶GL(g) such that, for all a∈G$ad_{a}:g⟶g$ is the *linear isomorphism* defined by $ad_{a}=d{\left(R_{a}^{-1}\circ L_{a}\right)}_{e}$. If we now assume *B* to be negative-definite, then -*B* is an inner product and is adjoint invariant. Thus, it is a classical result of the compact semi-simple Lie theory that -*B* induces a bi-invariant metric on *G*.

The Lie algebra of SO(n) is the set of skew-symmetric matrices which verifies $M^{T}=-M$. The Killing form on SO(n) is given by $B_{\mathrm{so}(n)}=\left(n-2\right)tr\left(XY\right)$, and as a result of the skew symmetry, we have $-B_{\mathrm{so}(n)}=\left(n-2\right)tr\left(XY^{T}\right)$. Therefore, it induces a bi-invariant metric and the previous formula can be plugged into the expression of the metric on the space of curves. In the sequel, the manifold that supports the curves is SO(n) endowed with its bi-invariant metric.

### 3.2. Basic Outline of Geometry

**Figure 2:**
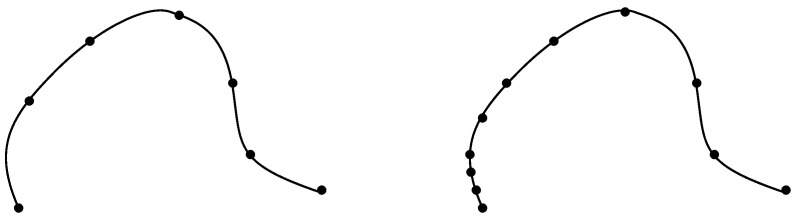
Example of a reparametrization of a curve. Here, it consists in changing the discretization with nonlinear time sample.

Curves of interest are those living in the Lie group of real rotation matrices; this yields c:[0,1]→SO(n). For the sake of clarity, assume that $c\in C^{\infty }\left(\left[0,1\right],SO\left(n\right)\right)$ , we will come back to the case of discrete curves later. To study the geometrical features of such curves, we interest ourselves with the set of all curves lying in SO(n) (where SO(n) is seen as a manifold) with nonvanishing velocity, i.e., $M=\left\{c\in C^{\infty }\left(\left[0,1\right],SO\left(n\right)\right):c^{\prime }\left(t\right)\neq 0\;\forall t\right\}$, this is in fact a sub-manifold of $C^{\infty }\left(\left[0,1\right],SO\left(n\right)\right)$. A curve *c* is thus a particular point in M. The tangent space at a curve *c* is given by

(23) $$T_{c}M=\left\{v\in C^{\infty }\left(\left[0,1\right],TSO\left(n\right)\right):v\left(t\right)\in T_{c(t)}SO\left(n\right)\right\}$$

where TSO(n) denotes the tangent bundle of the base manifold SO(n). Note that a tangent vector is a curve in the tangent space of SO(n) (see Theorem 5.6 in [51]). When comparing two curves, it is natural that the distance between these two curves should remain the same if the curves are only reparametrized, that is, if we define other curves that pass through the same points than the original curves but at different speeds. When the curve is discretized as we will see in the sequel, doing a reparametrization is equivalent to changing the chosen points (see Figure 2). A reparametrization is represented by increasing diffeomorphism ϕ∈D:[0,1]→[0,1] acting on the right of the curve by composition. In other words, we required that the Riemannian metric *g* on M satisfies the following property:

(24) $$g_{c\circ \phi }\left(u\circ \phi ,v\circ \phi \right)=g_{c}\left(u,v\right)$$

for all c∈M, $u,v\in T_{c}M$ and ϕ∈D.

This property is called reparametrization invariance. We insist on the fact that *g* is the metric on M, the space of all curves on SO(n) and not on SO(n) itself. In terms of distances, this gives

(25) $$d_{M}\left(c_{0}\circ \phi ,c_{1}\circ \phi \right)=d_{M}\left(c_{0},c_{1}\right)$$

where $d_{M}$ denote the distance on M corresponding to the metric *g*. The reparametrization introduced above induces an equivalence relation between points in M such that

(26) $$c_{0}∼c_{1}\Leftrightarrow \exists \phi \in D:c_{0}=c_{1}\circ \phi .$$

with this equivalence relation, a quotient space can be constructed as the collection of equivalence classes; it is named the shape space and has the following writing:

(27) S=M/∼,orS=M/D.

A distance function on the shape space is obtained from the distance on M as follows:

(28) $$d_{S}\left(\left[c_{0}\right],\left[c_{1}\right]\right)=\underset{\phi \in D}{inf}d_{M}\left(c_{0},c_{1}\circ \phi \right)$$

where $\left[c_{0}\right]$ and $\left[c_{1}\right]$ are representatives of the equivalence classes of $c_{0}$ and $c_{1}$ respectively. It can be shown that this distance is independent of the choice of the representatives. Some precautions has to be taken here: whereas M is a submanifold of the Fréchet manifold $C^{\infty }\left(\left[0,1\right],SO\left(n\right)\right)$, has proven by [52], Theorem 10.4, the shape space S is not a manifold and the principal bundle structure π=M→S is not formally defined. However, a manifold structure can be obtained if we only consider free immersion [53]. As the metric defined on the shape space is reparametrization-invariant, it is constant along the “fibers” (the origin point is fixed). Further explanations on the Riemannian submersion can be found in [54]. Closed curves being of main interest in this work, we can also define the set

(29) $$M^{c}=\left\{c\in C\left(\left[0,1\right],SO\left(n\right)\right):c^{\prime }\left(t\right)\neq 0,c\left(0\right)=c\left(1\right)\right\}.$$

Basically, the closure of a curve just imposes the equality of the first and the last point of it, and not of their first derivative. Consequently, $M^{C}$ turns into

(30) $$M^{c+}=\left\{c\in C\left(\left[0,1\right],SO\left(n\right)\right):c^{\prime }\left(t\right)\neq 0,c\left(0\right)=c\left(1\right),c^{\prime }\left(0\right)=c^{\prime }\left(1\right)\right\}.$$

We need now to introduce the Square Root Velocity function (SRV function) [55], in which a curve is represented by its starting point and its normalized velocity at each time *t*. There are several possibilities to define the SRV of a curve. The more general definition is the following

(31) $$\begin{array}{cc} F:M & \to SO(n)\times TM\\ c & \to \left(c\left(0\right),q=\frac{c^{\prime }}{\sqrt{||c^{\prime }||}}\right). \end{array}$$

However, we can go further and benefit from the specific case of the Lie group. In this section, we will denote the base manifold G=SO(n) to emphasize its group structure, and *g* an element of the group. As in [47], we consider only curves that start at the identity; this is because other curves can be reduced to this case by right or left translation. In these settings, it is interesting to turn the SRV function into the Transported SRV function (TSRV). This is basically the SRV that has been parallel transported to a reference point. Different versions have been given in [47,48,56] which differ in the choice of their reference point. For our case of study, the identity is our natural curve starting point and is thus a particularly good choice for being the reference point. In a Lie group, a parallel transport operation can be defined, here again, by the right (or left) translation. This justifies that we can take, as suggested in [47], a TSRV function of the following form:

(32) $$\begin{array}{cc} F_{Lie}:C^{\infty }\left(\left[0,1\right],G\right) & ⟶SO\left(n\right)\times \left\{q\in C^{\infty }\left(\left[0,1\right],g\right),q\left(t\right)\neq 0,\;\forall t\in \left[0,1\right]\right\}\\ F_{Lie}\left(c\right)\left(t\right) & =\left(c\left(0\right),q\left(t\right)\right)=\left(c\left(0\right),\frac{R_{c(t)*}^{-1}\left(c^{\prime }\left(t\right)\right)}{\sqrt{||c^{\prime }\left(t\right)||}}\right)=\left(c\left(0\right),\frac{T_{c}^{c(t)\to I}\left(c^{\prime }\left(t\right)\right)}{\sqrt{||c^{\prime }\left(t\right)||}}\right), \end{array}$$

where g is the Lie algebra, *R* is the right translation on the group, $R_{g_{1}}\left(g_{2}\right)=g_{2}g_{1}$, $R_{g*}=T_{e}R_{g}$ is the tangent map at the identity, ∣∣·∣∣ is a norm induced by a right-invariant metric on *G*, and $T_{c}^{c(t)\to I}$ denotes the parallel transport from c(t) to the identity according to the curve *c*. A curve is now represented pointwise as an element of the tangent bundle $\left(c(0),q(t)\right)\in M\times g$ (recall that *q* draws a curve in the tangent bundle), and c(0) is the identity element of the Lie group. The inverse of the SRV function is then straightforward: for every $q\in C^{\infty }\left(\left[0,1\right],TM\right)$, there exists a unique curve *c* such that $F\left(c_{i}\right)=q_{i}$ and $c\left(t\right)=\int_{0}^{t}q(r)||q(r)||dr$ where ∣∣·∣∣ is the norm in SO(n).

### 3.3. Metric and Distance over M and S

We now give insights on a relevant metric that should be used on M to compare different closed trajectories. The following development and expression of metrics and distances can be found in [45]. The distance on the shape space is used to compare how the curves are intrinsically different. It has been seen in [57] that the simple $L^{2}$ metric on M given by

(33) $$g_{c}^{L^{2}}\left(u,v\right)=\int \langle u,v\rangle ||c^{\prime }\left(t\right)||dt$$

where 〈·,·〉 is the Riemannian metric on SO(n), induced a vanishing metric on the shape space, that is, we cannot differentiate shape with this metric. To overcome this difficulty, the family of *elastic metric*, derived from the Sobolev metric [58,59], has been investigated for it is non-vanishing on the shape space. In the case of a Euclidean space $R^{n}$, it admits the expression:

(34) $$g_{c}^{a,b}\left(u,v\right)=\int \left(a^{2}\langle D_{l}u^{N},D_{l}v^{N}\rangle +b^{2}\langle D_{l}u^{T},D_{l}v^{T}\rangle \right)||c^{\prime }\left(t\right)||dt,$$

where $D_{l}u=h^{\prime }/||c^{\prime }||$, $D_{l}u^{T}=\langle D_{l}u,w\rangle w$, with $w=c^{\prime }/||c^{\prime }||$ and $D_{l}u^{N}=D_{l}u-D_{l}u^{T}$. Here, we are only interested in the special metric that has been proposed in [45], and which is an adaptation of the *elastic metric* for the Riemannian manifold. In our case this gives:

(35) $$g_{c}\left(u,v\right)=\int \left(\langle \nabla_{l}u^{N},\nabla_{l}v^{N}\rangle +\frac{1}{4}\langle \nabla_{l}u^{T},\nabla_{l}v^{T}\rangle \right)||\left(c^{\prime }t\right)||dt,$$

where ∇ is the Levi-Civita connection that corresponds to 〈·,·〉; $\nabla_{l}u=\frac{1}{||c^{\prime }||}\nabla_{c^{\prime }}h$, $\nabla_{l}u^{T}=\langle \nabla_{l}u,w\rangle w$, $w=c^{\prime }/||c^{\prime }||$. For the computations being done now in a manifold space, the Levi-Civita connection replaced the ordinary derivative of $R^{n}$.

**Figure 3:**
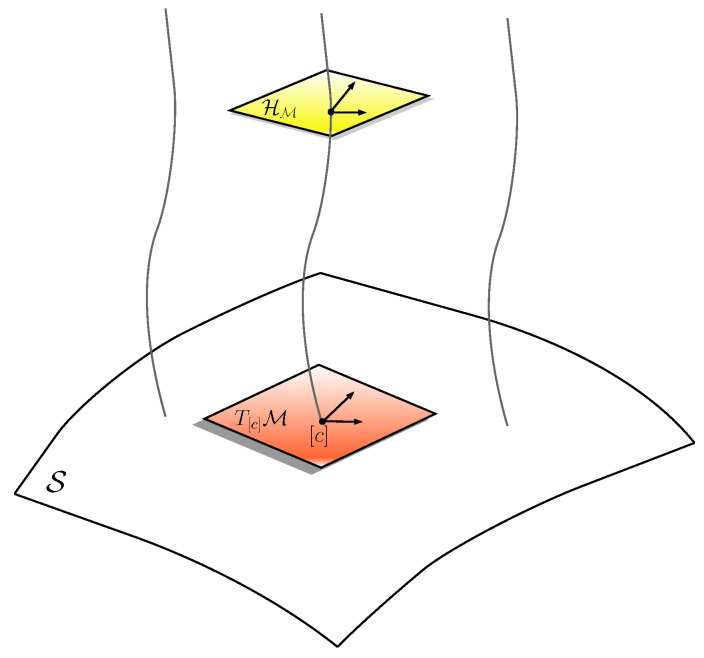
The tangent space $T_{\left[c\right]}M$ at a point [c] in the shape space S is isomorphic to the horizontal part $H_{M}$ of the tangent space at a point on the associated fiber.

Once geometry has been settled in M, the geometry of the shape space can be derived from its quotient structure. Let the tangent bundle be decomposed into a vertical and a horizontal subspace: $TM=H_{M}⊕V_{M}$, with $V_{M}=ker\left(T_{c}\pi \right)$ and $T_{c}$ the tangent map, π:M→S the principal bundle, and $H_{M}={\left(V_{M}\right)}^{⊥}$, see Figure 3. This metric is reparametrisation invariant, that is, constant along the fibers, hence we have

(36) $$g_{c}\left(u_{H},v_{H}\right)={\left[g\right]}_{\pi (c)}\left(T_{c}\pi \left(u\right),T_{c}\pi \left(v\right)\right)$$

where [g] denotes the metric *on the shape space*.

A similar result in a different (but still close) context is used in Lemma 1 of [60]. In terms of distances, this can be understood in the following sense. The geodesic s↦[c](s) between $\left[c_{0}\right]$ and $\left[c_{1}\right]$ in the shape space is the projection of the horizontal geodesic linking $c_{0}$ to the fiber containing $c_{1}$. In fact, the horizontal geodesic between $c_{0}$ of $c_{1}$ intersects the fiber at $c_{1}$ at the reparametrized version of $c_{1}$, $c_{1}\circ \phi$ which gives the distance in the shape space:

(37) $$\left[d\right]\left(\left[c_{0}\right],\left[c_{1}\right]\right)=d_{g}\left(c_{0},c_{1}\circ \phi \right)$$

where [d] denotes the distance in S, and $d_{g}$ denotes the distance on the space of curves induced by the aforementioned Riemannian metric. In the TSRV formulation, the distance problem of Equation (37) yields an optimisation problem:

(38) $$\left[d\right]\left(\left[c_{0}\right],\left[c_{1}\right]\right)=\underset{\phi \in D}{inf}{\left(\int_{0}^{1}||q_{0}\left(t\right)-q_{1}\left(\phi \left(t\right)\right)\sqrt{\phi^{\prime }\left(t\right)}|{|}^{2}\right)}^{1/2},$$

which is solved by a traditional gradient descent algorithm or a dynamic linear programming [47]. Finally, we have to mention that in a practical situation, the above formula has to be discretized. This is the object of [46]. Formulae are essentially similar, but in this setting, a curve is now represented by a set of points $c_{disc}\left(x_{0},x_{1},\cdots ,x_{n}\right)$ and the tangent space turns into

(39) $$T_{disc}M=\left\{v=\left(v_{0},v_{1},\cdots ,v_{n}\right),v_{i}\in T_{x_{i}}SO\left(n\right),\;\forall i\right\}.$$

Concerning the metric on the space of curves, it becomes

(40) $$g_{c_{disc}}\left(u,v\right)=\langle u_{0},v_{0}\rangle +\frac{1}{n}\sum_{i=0}^{n-1}\langle \nabla_{\partial c/\partial s}q^{u}\left(0,\frac{k}{n}\right),\nabla_{\partial c/\partial s}q^{v}\left(0,\frac{k}{n}\right)\rangle \;\forall u,v\in T_{disc}M$$

where, as before, for a $u\in T_{c_{disc}}M$, we define a path of piecewise geodesic curves $\left(s,t\right)\mapsto c^{u}\left(s,t\right)$ such that the following traditional initial conditions are fulfilled

$$\begin{array}{ccc} c^{u}\left(0,\frac{k}{n}\right) & = & x_{k},\;\mathrm{and}\;\\ \left(\partial c^{u}/\partial t\right)\left(0,\frac{k}{n}\right) & = & n\log_{x_{k}}\left(x_{k+1}\right). \end{array}$$

This is the discrete analogue of the tangent vector of a continuous curve at time *t*. The log function is the inverse of the exponential map on the base manifold, SO(n) for us, and here $c^{u}\left(s,\cdot \right)$ must be a geodesic on SO(n) between $x_{k/n}$ and $x_{(k+1)/n}$. The SRV function that appears in the formula refer to the SRV function of the piecewise geodesics $c^{u}\left(s,\cdot \right)$. Then, the discretized version of the SRV function, $q_{k}=\sqrt{n}\;\log_{x_{k}}\left(x_{k+1}\right)/\sqrt{||\log_{x_{k}}\left(x_{k+1}\right)||}$ is such that

(41) $$\nabla_{\partial c/\partial s}q\left(s,\frac{k}{n}\right)=\nabla_{\partial c/\partial s}q_{k}\left(s\right)$$

### 3.4. The Geodesic Equation

**Figure A1:**
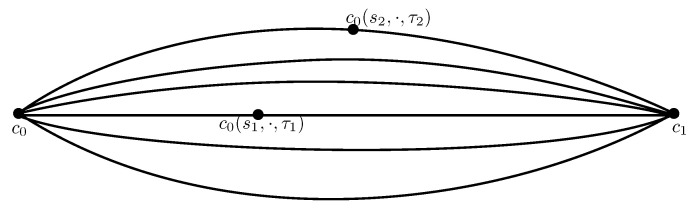
we consider a beam of curves, which consists in a slight modification of the geodesic. The different curves are indexed by τ. The idea is to find which of these curves gives the minimal energy to go from $c_{0}$ to $c_{1}$.

Let us now give the geodesic equation, relative to our chosen measure. As a result of the TSRV, the geodesic equation takes a much simpler form than what can be found in [45,46]. The formula can be found in [47]. For the sake of completeness, we give a reformulated proof in Appendix B. Recall that a geodesic is a particular path of curves. A path of curves is a continuous set of curve s↦c(s,·) such that for each *s*, c(s,·) is a point in M, or, equivalently, a curve in *M*, (see Figure A1). Thus, for each curve of the path of curves, we can defined its TSRV function. Then for all s∈[0,1], we have (we omit the letter `*s*’ for clarity): $q=\frac{T_{c}^{c(t)\to I}\left(\partial c/\partial t\right)}{\sqrt{||\partial c/\partial t||}}$

**Theorem** **3.** *A path of curves $\left[0,1\right]∋s\mapsto \left(c(s,0),q(s,t)\right)$ (t is the parameter of the curve c(s,·))is a geodesic on M if and only if*

(42) $$\nabla_{\partial c/\partial s}\left(\nabla_{\partial c/\partial s}q\left(s,t\right)\right)\left(s,t\right)=0\;\forall s,t$$

**Proof.**  Appendix B ☐

Thus, we have a quite familiar expression for the geodesic interpolation between two curves $c_{0}$ and $c_{1}$, expressed in their TSRV domain:

(43) $$F_{Lie}^{-1}\left(\left(1-s\right)F_{Lie}\left(c_{0}\right)+sF_{Lie}\left(c_{1}\right)\right)$$

for s∈[0,1]. This expression is nothing but a linear interpolation on the transported tangent space.

We now have all the ingredients to give the procedure for nonstationary processes characterization and comparison:

1. **Input**: a set of rotation matrices ${\left\{W_{i}\right\}}_{i}$, seen as a partial observation of a closed trajectory on SO(n).
2. Map the set of matrices $\left\{W_{i}\right\}$ into the a set of matrices in the Lie algebra $\left\{V_{i}\right\}$ using the inverse exponential map.
3. Interpolate with splines between matrices $V_{i}$ [61,62].
4. Go back in the base manifold SO(n) with the exponential map.
5. Shift the interpolated curve in order to fulfill the condition c(0)=e and compute the SRV transformation given by (41)
6. Compute the distance defined by (38). The optimization is carried out by dynamic programming.
7. **Output**: distance between two curves in the manifold defined by the set of curves in SO(n), and geodesic path between the curves.

The interpolation computations are carried out in the Lie algebra, which is a vector space, and thus it does not demand great computational resources. The discretization step, which amounts to choosing certain values among the continuous curve, is also done in the Lie algebra. In this way, we avoid the calculation of matrices exponential that would have been discarded at the end. We finally note that geodesic shooting [45,63] or other path straightening methods could be applied to obtain a geodesic path between two curves, and between the shapes of the two curves.

### 3.5. Results

**Figure 4:**
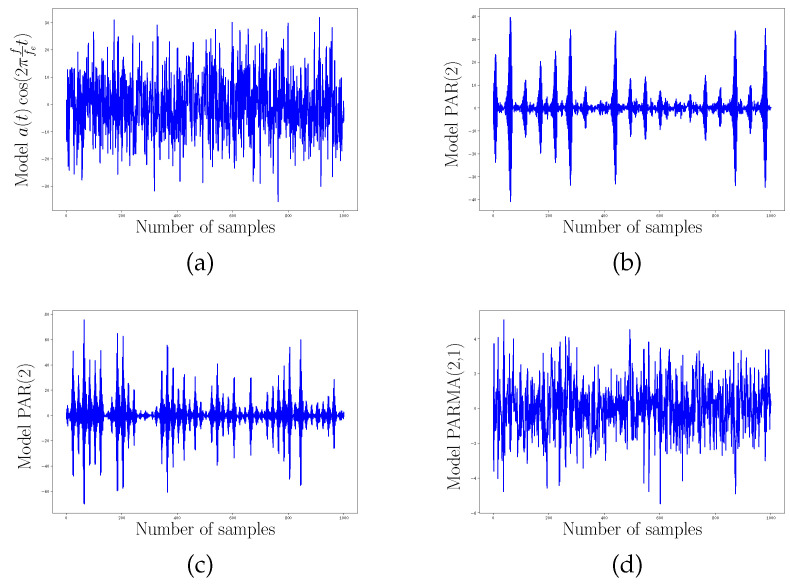
1000 samples of PC processes generated by (**a**) a modulated zero mean and unit variance stationary random process a(t); (**b**) a periodic AR(2) model with a period of 54 points; (**c**) a periodic AR(2) model with a period of 20 points; and (**d**) a periodic ARMA(2,1) model with a period of 20 points.

**Figure 5:**
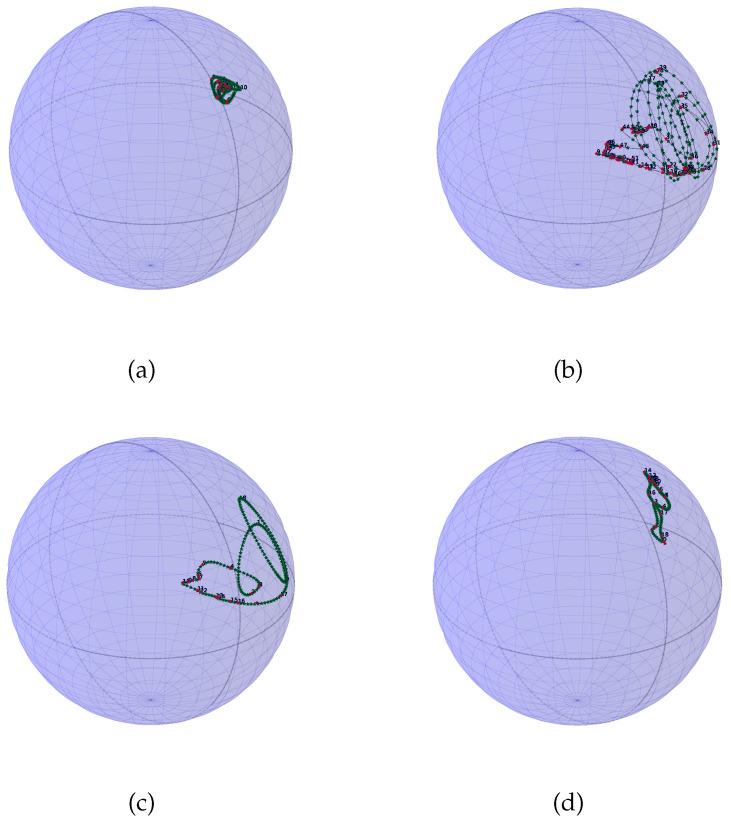
Representation inside the ball of radius π of the four PC processes drawn in Figure 4, arranged in the same order with 200 interpolated points represented with green stars, the dashed black line is the theoretical curve and the red dots are the representation of dilation matrices. (**a**) is the representation of Figure 4a, (**b**) is the representation of Figure 4b, (**c**) is the representation of Figure 4c and (**d**) is the representation of Figure 4d.

**Figure 6:**
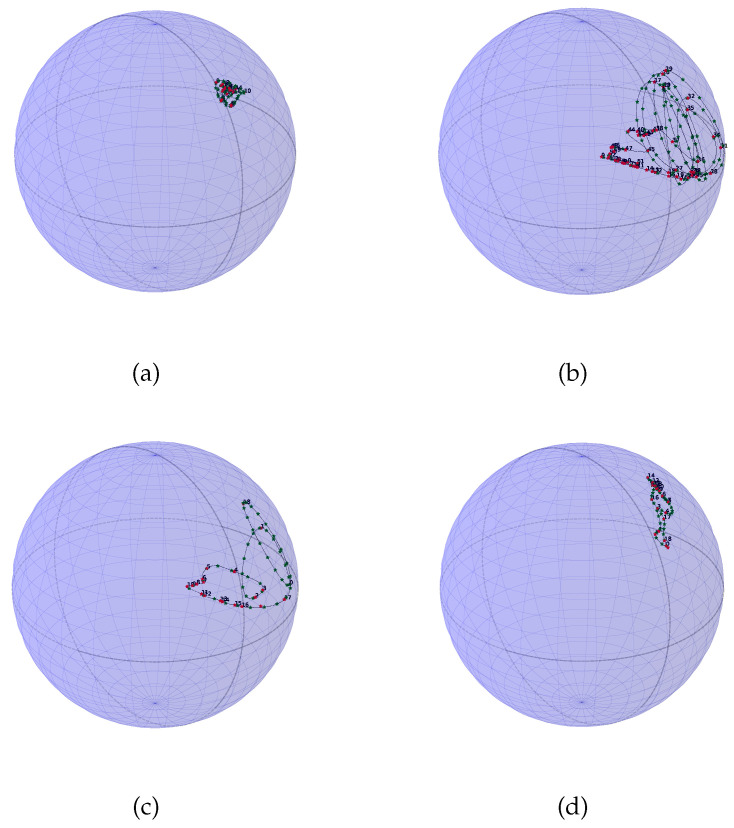
Representation inside the ball of radius π of the four PC processes drawn in Figure 4, arranged in the same order with 50 interpolated points except for plot (**b**) which has been computed with 100 interpolated points represented with green stars; the dashed black line is the theoretical curve and the red dots are the representation of dilation matrices. (**a**) is the representation of Figure 4a, (**b**) is the representation of Figure 4b, (**c**) is the representation of Figure 4c and (**d**) is the representation of Figure 4d.

**Figure 7:**
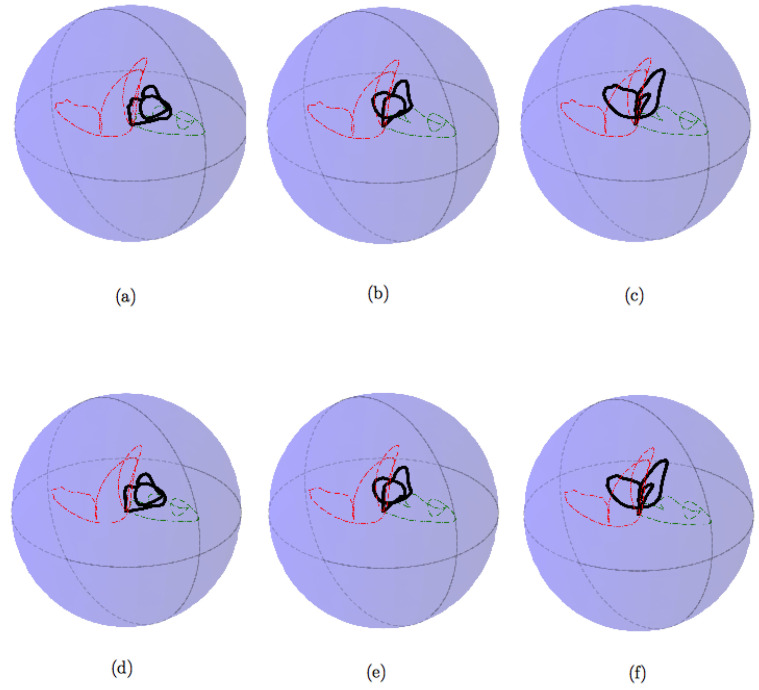
Geodesic interpolation with respect to (43) between the green dashed curve (gold standard signal of Figure 8) and the dashed red curve (signal of Figure 4c), first row for 200 interpolated points, second row for 50 interpolated points. For this scenario s∈[1/4,1/2,3/4] which corresponds to [(**a**–**d**), (**b**–**e**), (**c**–**f**)] respectively.

In order to expose how the approach of this work gives interesting results for PC processes understanding, we propose to compare four PC processes, displayed along with Figure 4. We also bring their corresponding SO(3) representation on Figure 5 and Figure 6 with 200 interpolated points and 50 interpolated points respectively. For this scenario we have generated four PC processes with 1000 samples each. A classical amplitude modulated model $a\left(t\right)\cos\left(2\pi f/f_{e}\;t\right)$ where a(t) is a zero mean and unit variance stationary random process with a period of 20 points, a periodic AR(2) with a period of 20 points, a periodic AR(2) (AutoRegressive) with a period of 54 points, and a periodic ARMA(2,1) (AutoRegressive Moving Average) with a period of 20 points have been generated. We have used the R package PerARMA to generate the periodic ARMA and AR signals and we finally used the PerPACF function of this package to estimate the 20 (or 54) sequences of three parcors each. The analysis of Figure 4 with Figure 5 shows that the spectral measure of the amplitude modulated signal of Figure 4a has dilation matrices which do not spread a lot; we could think that this process is almost stationary due to the weak distance between each matrices. A contrario, whereas the temporal form of the PARMA(2,1) signal of Figure 4d is quite identical to the amplitude modulated signal of Figure 4a, their representation on SO(3) is very different. The spectral measure of the PARMA(2,1) signal spread much more. Lastly, when we observe the Figure 4b,c which are generated with the same model but with a different period, we can see that the more the number of points per period is important, the more the curve wraps. We also note one of the advantages of using spline interpolation. Along with Figure 5, we can remark that the curvature is well approximated owing to more and closer interpolated points. For this figure, we had approximatively four times more interpolated points than original ones whereas for Figure 6 we computed roughly twice more interpolated points than the original ones. Actually, for such a number, a change in the grid could be thought but it seems again that the spline interpolation gives good points repartition. This is mainly illustrated by Figure 7 for which the difference between geodesic interpolation of the curve associated with the periodic AR(2) signal of Figure 4c for 200 interpolated points and 50 interpolated points is very weak.

**Figure 8:**
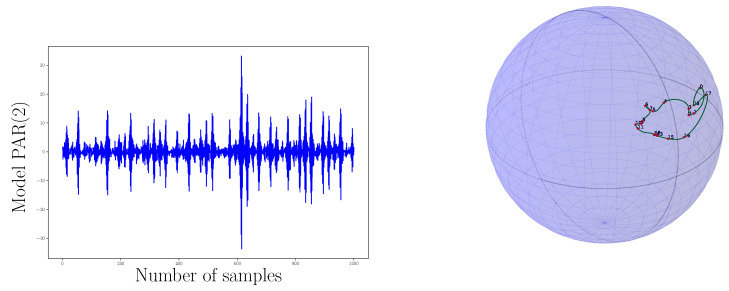
A PAR(2) signal with a period of 20 points, 1000 samples were generated, and its corresponding SO(3) representation inside the ball of radius π.

To end this analysis by the example, we have computed the distance defined by (38) between the PC process of Figure 8 and all the PC processes studied and displayed on Figure 4 and Figure 5. The distances are reported inside Table 1. Clearly, the distances between the shapes of the curves characterizing the spectral measure of each PC process, reveal some spectral proximity between the PC processes benchmarked. We have gray colored the row of the PAR(2) signal model indexed by letter (c). Whatever the number of interpolated points and the dimension of the base manifold, the spectral representation through dilation operators of this signal is the nearest on SO(3) and the second nearest for SO(4) and SO(5) to the PAR(2) signal of reference. The second interesting signal is the one indexed by letter (b) and stands for a PAR(2) signal with exactly the same model parameters as that of the signal of reference but with 54 points of periodicity instead of 20. We have lightly gray colored its associated row on Table 1 when it had the shortest distance. The curve associated with this signal has many wraps on its representation, and it has consequently the greatest distance on SO(3), but increasing the dimension improves the comparison. Indeed, it finally has the shortest distance on SO(4) and SO(5). This is particularly interesting to see that there is a competition between the curves of a PAR(2) with different model parameters but the same periodicity and a PAR(2) with the same model parameters but different periodicity.

We end by noticing that the PARMA(2,1) has the second shortest distance to the PAR(2) signal model of reference on SO(3). As Figure 5 shows, their spectral measure evolves in a similar way with one major loop and a second less important loop. However, once the dimension of the base manifold increases, the assumption that the two processes may be close is strongly rejected by the fact that the PARMA(2,1) signal model has the longest distance. Finally, these observations leave open besides the question of the topology of these curves and how it could be used for the classification.

## 4. Conclusions

We have introduced a new vision of stochastic processes through geometry induced by dilation. The dilation matrices of given processes were obtained by a composition of rotations whose angles correspond to the well-known parcors, reflexion coefficients or Verblunski coefficients. The advantage of working with these particular matrices is that they are strongly related to the stochastic measure of the process, and thus, to its spectra. Furthermore, the dilation theory is independent of the stationarity of the underlying process; when the signal is stationary, its dilation operator is related to the Naimark dilation whereas when the signal is nonstationary, a set of dilation matrices is obtained and it is related to the Kolmogorov decomposition. Rigorously, dilation matrices are infinite dimensional, although we turn them into rotation matrices by truncation. Each of them belongs to the Special Orthogonal Group SO(n) or the Special Unitary Group SU(n) depending on the real- or complex-valued process under study. We focused our attention on the Periodically Correlated (PC) class of nonstationary processes for which a timely ordered set of dilation matrices describes the process measure. This set draws a closed curve on the Lie group of rotation matrices, and describing or classifying the different PC processes is made by curves comparison. We use for that the Square Root Velocity (SRV) function which represents a curve by its starting point and by its normed velocity vector on the space or curves. The metric in the space of curve naturally extends to the space of shapes. It is then possible to compare the shape of curves when the metric is translated into the Lie algebra, achieving therefore a closed-form expression and easy computation. Nonstationary processes are then characterized via their embedded curves.

## Figures and Tables

**Table 1:** Table of the distances between all the PC processes of Figure 4 to the gold standard PC process of Figure 8 through the distance of their curves’ shapes on SO(3), SO(4) and SO(5). We have interpolated with roughly twice and four times the number of original points. We also applied here a DP to solve the optimization assignment problem.

Model of Signal Displayed in Figure 4	Distance to the Signal of Figure 8					
	SO(3)		SO(4)		SO(5)	
	200 pts	50 pts	200 pts	50 pts	200 pts	50 pts
(a)	5.72	4.47	97.19	26.86	526.36	95.47
(b)—100 pts instead of 50 pts	31.63	28.98	41.78	12.98	298.64	220.32
(c)	3.44	3.29	90.89	20.23	476.55	116.06
(d)	4.!9	4.50	187.42	50.36	621.51	171.73

## Appendix A. Defect Operator, Elementary Rotation

Introducing the *defect operator* of a contraction *T* as being $D_{T}={\left(I-T^{*}T\right)}^{1/2}$, we have the following factorisation:

(A1) $$\left(\begin{array}{cc} X & Y\\ Y^{*} & Z \end{array}\right)=\left(\begin{array}{cc} X^{1/2} & 0\\ Z^{1/2}\Gamma^{*} & Z^{1/2}D_{\Gamma } \end{array}\right)\left(\begin{array}{cc} X^{1/2} & \Gamma Z^{1/2}\\ 0 & D_{\Gamma }Z^{1/2} \end{array}\right)$$

where *X* and *Y* are positive matrices. Note that this is a Cholesky factorisation-type result. This type of decomposition is used as the square root of matrices in the construction of the dilation. A corollary is that the operator $\left(\begin{array}{cc} I & T\\ T^{*} & I \end{array}\right)$ is positive if and only if *T* is a contraction.

**Theorem** **A1.**
*Let X and Y be operators in z. The following statements are equivalent:*

- *There exists a contraction *Γ* in z such that X=ΓY,*
- *$X^{*}X⩽Y^{*}Y$.*

**Proof.** This result can be proved by taking the contraction Γ with respect to ΓXh=Yh. [41]. ☐

As a corollary If, $X^{*}X=Y^{*}Y$, then there exists a partial isometry *V* such that VX=Y. It is easy to see that we can choose *V* to be the contraction Γ defined above. Isometry *V* can also be assumed unitary. For a positive operator A∈L(H), if we denote by $A^{1/2}$ its unique positive square root, then every *L* such that $L^{*}L=A$ is related to $A^{1/2}$ by $A^{1/2}=VL$ (or $A^{1/2}=L^{*}V^{*}$).

Let us state another theorem that intervenes much in Constantinescu’s factorisation of positive-definite kernel. Note that in the following, R(Γ) will denote the close range of the operator Γ. We start with a basic case:

**Theorem** **A2** (row contraction)**.**
*Let $T=\left[T_{1}\;T_{2}\right]\;\in L\left(H_{1}⊕H_{2},H\right)$, then ∣∣T∣∣⩽0 if and only if there exists contractions $\Gamma_{1}\in L\left(H_{1},H\right)$ and $\Gamma_{2}\in L\left(H_{2},H\right)$ such that*

(A2) $$T=[\Gamma_{1}\;D_{\Gamma_{1}^{*}}\Gamma_{2}]$$

**Proof.** The proof is a simple application of Theorem A1. For the if part, it is obvious that we can take $\Gamma_{1}$ to be $T_{1}$. Then ∣∣T∣∣⩽1 implies

(A3) $$I-TT^{*}=I-\Gamma_{1}\Gamma_{1}^{*}-T_{2}T_{2}^{*}⩾0$$

with $D_{\Gamma_{1}^{*}}^{2}⩾T_{2}T_{2}^{*}$. Hence, there exists Δ such that $\Delta D_{\Gamma_{1}^{*}}=T_{2}^{*}$. Choosing $\Gamma_{2}=\Delta^{*}$ finishes the argument. ☐

In the same way as that of the Cholesky factorisation, we can write down the defect operator for the whole contraction $T=[T_{1}\;T_{2}]$ [41] to be

(A4) $$D_{T}^{2}=\left(\begin{array}{cc} D_{\Gamma_{1}} & 0\\ -\Gamma_{2}^{*}\Gamma_{1} & D_{\Gamma_{1}} \end{array}\right)\left(\begin{array}{cc} D_{\Gamma_{1}} & -\Gamma_{1}^{*}\Gamma_{2}\\ 0 & D_{\Gamma_{1}}. \end{array}\right)$$

Therefore, with Theorem A2, we have an operator α such that

(A5) $$D_{T}=\left(\begin{array}{cc} D_{\Gamma_{1}} & 0\\ -\Gamma_{2}^{*}\Gamma_{1} & D_{\Gamma_{1}} \end{array}\right)\alpha$$

Similarly,

(A6) $$D_{T^{*}}^{2}=\left(D_{\Gamma_{1}^{*}}D_{\Gamma_{2}^{*}}D_{\Gamma_{2}^{*}}D_{\Gamma_{1}^{*}}\right)$$

and the general case is:

**Theorem** **A3** (Structure of row contraction)**.**
*The following are equivalent:*

- *The operator $T^{n}=\left[T_{1}\;T_{2}\;\cdots T^{n}\right]$ in $L({⊕}_{k=1}^{n}H_{k},H^{\prime })$ is a contraction*
- *$T_{1}=\Gamma_{1}$ is a contraction and, for k>2, there exists uniquely determined contractions $\Gamma_{k}\;\in L\left(H_{k},R\left(\gamma_{k}\right)\right)$ such that $T_{k}=D_{\Gamma_{1}^{*}}D_{\Gamma_{2}^{*}}\cdots D_{\Gamma_{k-1}^{*}}\Gamma_{k}$.*

Furthermore, the defect operators of the whole contraction *T* are of the form

(A7) $$\begin{array}{cc} D_{T}^{2}= & \\ & \left(\begin{array}{cccc} D_{\Gamma_{1}} & 0 & \cdots & 0\\ -\Gamma_{2}^{*}\Gamma_{1} & D_{\Gamma_{2}} & \cdots & 0\\ \vdots & \vdots & \ddots & \vdots \\ -\Gamma_{n}^{*}D_{\Gamma_{n-1}^{*}}\cdots D_{\Gamma_{2}^{*}} & -\Gamma_{n}^{*}D_{\Gamma_{n-1}^{*}}\cdots D_{\Gamma_{3}^{*}}\Gamma_{2} & \cdots & D_{\Gamma_{n}} \end{array}\right)\left(\begin{array}{cccc} D_{\Gamma_{1}} & -\Gamma_{1}^{*}\Gamma_{2} & \cdots & -\Gamma_{1}^{*}D_{\Gamma_{2}^{*}}\cdots D_{\Gamma_{n-1}^{*}}\Gamma_{n}\\ 0 & D_{\Gamma_{2}} & \cdots & -\Gamma_{2}^{*}D_{\Gamma_{3}^{*}}\cdots D_{\Gamma_{n-1}^{*}}\Gamma_{n}\\ \vdots & \vdots & \ddots & \vdots \\ 0 & 0 & \cdots & D_{\Gamma_{n}} \end{array}\right) \end{array}$$

and

(A8) $$D_{T^{*}}^{2}=D_{\Gamma_{1}^{*}}\cdots D_{\Gamma_{n}^{*}}D_{\Gamma_{n}^{*}}\cdots D_{\Gamma_{1}^{*}}$$

**Proof.** It can be proved straightforwardly by induction. ☐

This construction permits to understand the apparition of the operators α and β in the publications of Constantinescu which are used to identify the defect space of the components (the underlying contractions of a row contraction) of a row contraction with the defect space of the row contraction itself. Same results are readily obtained for a column contraction of the form $T=\left(\begin{array}{c} T_{1}\\ \vdots \\ T_{2} \end{array}\right)$.

## Appendix B. Geodesic Equation in the Space of Curve M

To have a complete insight on the geodesic equation, we give the proof for a more general case that arises when considering the SRV and not only the TSRV function of a curve, that is, the curves are parametrised by their starting point and their velocity, but their starting points are not transported to the identity.

**Theorem** **A4.**
*A path of curves $\left[0,1\right]∋s\mapsto \left(c(s,0),q(s,t)\right)$ (t is the parameter of the curve c(s,·))is a geodesic on M if and only if:*

(A9) $$\begin{array}{cc} \nabla_{\partial c/\partial s}c\left(s,0\right)+\int_{0}^{1}R\left(q\left(s,t\right),\nabla_{\partial c/\partial s}q\left(s,t\right)\right)\left(c\left(s,0\right)\right)dt & =0\;\forall s \end{array}$$

(A10) $$\begin{array}{cc} \nabla_{\partial c/\partial s}\left(\nabla_{\partial c/\partial s}q\left(s,t\right)\right)\left(s,t\right) & =0\;\forall s,t \end{array}$$

Similarly to [45,48], we consider a variation of the path s↦c(s,0),q(s,t) starting and ending at the same points, we denote $\left\{\left(c(s,0,\tau ),q(s,t,\tau )\right)\right\}$. In Figure A1, to get a clear picture, we have represented a variation of a path of curves with fixed starting and ending points. Although similar, the situation here is a bit different because of the representation of the curve through its SRV function, which we can hardly represent. However, the process remains similar. We emphasise the subtle difference with [45]. Here, we work directly in the tangent space representation, via the SRV representation, and not with “the whole family” of curves c(s,t,τ).

We denote $\partial_{\tau }c\left(s,0,\tau \right)=\frac{\partial c(s,0,\tau )}{\partial \tau }$, and similarly for $\partial_{s}c\left(s,0,\tau \right)$ and $\partial_{\tau }c\left(s,0,\tau \right)$. The energy of the path indexed by τ is

(A11) $$E\left(\tau \right)=\frac{1}{2}\int_{0}^{1}\langle \partial_{s}c\left(s,0,\tau \right),\partial_{s}c\left(s,0,\tau \right)\rangle +\langle \nabla_{\partial c/\partial s}q\left(s,t,\tau \right),\nabla_{\partial c/\partial s}q\left(s,t,\tau \right)\rangle ds.$$

Recall that the derivative of the inner product is given by $\frac{d}{dx}\langle f\left(x\right),f\left(x\right)\rangle =2*\langle f\left(x\right),\frac{df}{dx}\rangle$. Then

(A12) $$E^{\prime }\left(0\right)=\int_{0}^{1}\langle \nabla_{\partial c/\partial \tau }\frac{\partial c}{\partial s}\left(s,0,0\right),\frac{\partial c}{\partial s}\left(s,0,0\right)\rangle +\langle \nabla_{\partial c/\partial \tau }\nabla_{\partial c/\partial s}q\left(s,t,0\right),\nabla_{\partial c/\partial s}q\left(s,t,0\right)\rangle ds$$

with $\nabla_{\partial c/\partial s}\left(\partial_{\tau }c\left(s,0,\tau \right)\right)=\nabla_{\partial c/\partial \tau }\left(\partial_{s}c\left(s,0,\tau \right)\right)$ and owing to the curvature tensor $R\left(\partial_{\tau }c\left(s,0,\tau \right),\partial_{s}c\left(s,0,\tau \right)\right)(q\left(s,t,\tau \right)=\nabla_{\partial c/\partial \tau }\nabla_{\partial c/\partial s}\left(q\left(s,t,\tau \right)\right)-\nabla_{\partial c/\partial s}\nabla_{\partial c/\partial \tau }\left(q\left(s,t,\tau \right)\right)$ we have

(A13) $$\begin{array}{c} E^{\prime }\left(0\right)=\\ \int_{0}^{1}\langle \nabla_{\partial_{s}c}\partial_{\tau }c\left(s,0,\tau \right),\partial_{s}c\left(s,0,\tau \right)\rangle +\langle R\left(\partial_{\tau }c\left(s,0,\tau \right),\partial_{s}c\left(s,0,\tau \right)\right)q\left(s,t,\tau \right),\nabla_{\partial_{s}}q\left(s,t,\tau \right)\rangle \\ +\langle \nabla_{\partial_{s}c}\nabla_{\partial_{\tau }c}q\left(s,t,0\right),\nabla_{\partial_{s}c}q\left(s,t,0\right)\rangle \;ds. \end{array}$$

Integrating by parts now, allows to have

$$\begin{array}{cc} \int_{0}^{1}\langle \nabla_{\partial_{\tau }c}\partial_{s}c\left(s,0,\tau \right),\partial_{s}c\left(s,0,\tau \right)\rangle ds & =-\int_{0}^{1}\langle \nabla_{\partial_{s}c}\partial_{s}c\left(s,0,\tau \right),\partial_{\tau }c\left(s,0,\tau \right)\rangle ds\\ \int_{0}^{1}\langle \nabla_{\partial_{s}c}\nabla_{\partial_{\tau }c}\left(q\left(s,t,\tau \right)\right),\nabla_{\partial_{s}}q\left(s,t,\tau \right)\rangle & =-\int_{0}^{1}\langle \nabla_{\partial_{s}c}\nabla_{\partial_{s}c}\left(q\left(s,t,\tau \right)\right),\nabla_{\partial_{\tau }}q\left(s,t,\tau \right)\rangle \end{array}$$

which yields to

(A14) $$\begin{array}{l} E^{\prime }\left(0\right)=\int_{0}^{1}\left(-\langle \nabla_{\partial_{s}c}\partial_{s}c\left(s,0,\tau \right),\partial_{\tau }c\left(s,0,\tau \right)\rangle \right)\\ \;+\langle R\left(\partial_{\tau }c\left(s,0,\tau \right),\partial_{s}c\left(s,0,\tau \right)\right)q\left(s,t,\tau \right),\nabla_{\partial_{s}}q\left(s,t,\tau \right)\rangle \\ \;+(-\langle \nabla_{\partial_{s}c}\nabla_{\partial_{s}c}q\left(s,t,0\right),\nabla_{\partial_{\tau }c}q\left(s,t,0\right)\rangle )ds, \end{array}$$

for any vector fields X,Y,Z,W, 〈R(X,Y)Z,W〉=−〈R(W),Z〉, we consequently obtain

(A15) $$\begin{array}{l} E^{\prime }\left(0\right)=-\int_{0}^{1}\langle \nabla_{\partial_{s}c}\partial_{\tau }c\left(s,0,\tau \right),\partial_{s}c\left(s,0,\tau \right)\rangle \\ \;+\langle R\left(q\left(s,t,\tau \right),\nabla_{\partial_{s}}q\left(s,t,\tau \right)\right)\left(\partial_{s}c\left(s,0,\tau \right)\right),\partial_{\tau }c(s,0,\tau \rangle \\ \;+\langle \nabla_{\partial c/\partial s}\nabla_{\partial c/\partial \tau }q\left(s,t,0\right),\nabla_{\partial c/\partial s}q\left(s,t,0\right)\rangle ds. \end{array}$$

Geodesic corresponds to minimal energy. It means that every other path that starts and ends at the same points should require more energy to travel than the geodesic. We then have to solve $E^{\prime }\left(0\right)=0$ for every $\partial_{\tau }c\left(s,0,\tau \right)$ and every $\nabla_{\partial_{\tau }}\left(q\left(s,t,\tau \right)\right)$. This gives the result.

Now when the framework is given by the TSRV and not by the SRV, only the second part of the geodesic equation remains as a result of the fixed starting point which corresponds to the identity element. This very much simplifies the equation, even though the derivation is the same.
